# Role of NRP1 in Bladder Cancer Pathogenesis and Progression

**DOI:** 10.3389/fonc.2021.685980

**Published:** 2021-06-23

**Authors:** Yang Dong, Wei-ming Ma, Zhen-duo Shi, Zhi-guo Zhang, Jia-he Zhou, Yang Li, Shao-qi Zhang, Kun Pang, Bi-bo Li, Wen-da Zhang, Tao Fan, Guang-yuan Zhu, Liang Xue, Rui Li, Ying Liu, Lin Hao, Cong-hui Han

**Affiliations:** ^1^ Department of Urology, Xuzhou Central Hospital, Xuzhou, China; ^2^ Medical College of Soochow University, Suzhou, China; ^3^ College of Life Sciences, Jiangsu Normal University, Xuzhou, China; ^4^ Department of Central Laboratory, Xuzhou Central Hospital, Xuzhou, China; ^5^ Nanjing University of Traditional Chinese Medicine, Nanjing, China

**Keywords:** NRP1, bladder cancer, proliferation, apoptosis, neovascularisation, migration, invasion

## Abstract

Bladder urothelial carcinoma (BC) is a fatal invasive malignancy and the most common malignancy of the urinary system. In the current study, we investigated the function and mechanisms of Neuropilin-1 (NRP1), the co-receptor for vascular endothelial growth factor, in BC pathogenesis and progression. The expression of NRP1 was evaluated using data extracted from GEO and HPA databases and examined in BC cell lines. The effect on proliferation, apoptosis, angiogenesis, migration, and invasion of BC cells were validated after *NRP1* knockdown. After identifying differentially expressed genes (DEGs) induced by *NRP1* silencing, GO/KEGG and IPA^®^ bioinformatics analyses were performed and specific predicted pathways and targets were confirmed *in vitro.* Additionally, the co-expressed genes and ceRNA network were predicted using data downloaded from CCLE and TCGA databases, respectively. High expression of NRP1 was observed in BC tissues and cells. *NRP1* knockdown promoted apoptosis and suppressed proliferation, angiogenesis, migration, and invasion of BC cells. Additionally, after *NRP1* silencing the activity of MAPK signaling and molecular mechanisms of cancer pathways were predicted by KEGG and IPA^®^ pathway analysis and validated using western blot in BC cells. *NRP1* knockdown also affected various biological functions, including antiviral response, immune response, cell cycle, proliferation and migration of cells, and neovascularisation. Furthermore, the main upstream molecule of the DEGs induced by *NRP1* knockdown may be *NUPR1*, and *NRP1* was also the downstream target of *NUPR1* and essential for regulation of *FOXP3* expression to activate neovascularisation. *DCBLD2* was positively regulated by *NRP1*, and PPAR signaling was significantly associated with low *NRP1* expression. We also found that NRP1 was a predicted target of miR-204, miR-143, miR-145, and miR-195 in BC development. Our data provide evidence for the biological function and molecular aetiology of NRP1 in BC and for the first time demonstrated an association between NRP1 and NUPR1, FOXP3, and DCBLD2. Specifically, downregulation of *NRP1* contributes to BC progression, which is associated with activation of MAPK signaling and molecular mechanisms involved in cancer pathways. Therefore, NRP1 may serve as a target for new therapeutic strategies to treat BC and other cancers.

## Introduction

Bladder urothelial carcinoma (BC), one of the most prevalent urologic malignancies worldwide, is refractory to many common treatments (1), and its incidence and mortality rate are the highest among genitourinary tumors in China (2). BC generally has a low cure rate and a high relapse rate. Although most cases are initially diagnosed as non-muscle invasive by pathological examination, discontinuation or delayed treatment due to lack of regular re-examination ultimately leads to muscle invasive BC with a great risk of distant metastasis (3). The 5-year survival rate of metastatic BC is approximately 5%, due to limited available therapies (4). Recently, multiple therapeutic approaches for BC have been explored, however, no obvious improvement has been reported in the overall survival rate. Therefore, novel targets and effective strategies for BC therapy are urgently needed.

Neuropilins (NRPs) are transmembrane glycoprotein receptors with a well-described role in interacting with the semaphorins and vascular endothelial growth factor family members (5). NRP1 encodes certain NRPs and participates in axon guidance and angiogenesis. NRP1 mutations result in fatal abnormalities in the cardiovascular system (6). Further, many studies have observed the abnormally high expression of NRP1 in multiple tumor types, including neuroblastomas and bile duct, gastric, pancreas, lung, prostate, breast, and colon cancers (5, 7). Previously, we have demonstrated that NRP1 was upregulated in patients with BC, correlating with poor prognosis (7). However, the molecular mechanisms underlying how NRP1 regulates the progression of BC remain unclear. Therefore, in the current study, we aimed to observe the regulation of *NRP1* silencing on proliferation, apoptosis, migration, and invasion in BC cells, and elucidate the potential signal pathways involved in the inhibition of BC progression after *NRP1* knockdown, as well as the potential mechanisms employed by NRP1 in BC pathogenesis and progression.

## Materials and Methods

### Cell Lines

The human bladder immortalized epithelium cell line SVHUC1 and BC cell lines including T24, 5637, J82, UMUC3 and RT4 were purchased from the Cell Resource Center of the Shanghai Institutes for Biological Sciences, Chinese Academy of Sciences (Shanghai, China). All cell lines were cultured as described previously (8). We cultured all cell lines in RPMI 1640 medium with 100 U/mL penicillin, 100 ug/mL streptomycin, and 10% foetal bovine serum at 5% CO2 in a 37°C humidified culture environment. Short-tandem repeat profiling was used to authenticate the cell lines less than 6 months before this project was initiated, and the cells were not in culture for more than 2 months.

### Data Mining and Collection

We downloaded three gene expression datasets [GSE3167 (9), GSE65635 (10), and GSE120736 (11)] from GEO (http://www.ncbi.nlm.nih.gov/geo) (12). All studies employed tissue samples gathered from human non-muscle invasive BC and muscle invasive BC tissues. The annotation information provided by the platform was referenced to convert the probes into the corresponding gene symbols. The Human Protein Atlas (HPA) database (https://www.proteinatlas.org/), was used to identify the protein expression of NRP1 in BC tissues (13). BC patients in TCGA cohorts were also included in the study. The relevant lncRNA expression and miRNA data and clinical data of BC were downloaded from TCGA Bladder Carcinoma (TCGA-BLCA) study of the official TCGA website (https://cancergenome.nih.gov/), updated on May 07, 2020. RNA expression (RNA-Seq) data of *NRP1* in different urinary tract cancer cell lines (n = 26) were obtained from the Cancer Cell Line Encyclopedia (CCLE) database (https://portals.broadinstitute.org/ccle/about) (14), updated on January 02, 2019.

### RNA Isolation and Quantitative Real-Time Reverse Transcription Polymerase Chain Reaction (qRT-PCR)

According to the manufacturer’s instructions, total RNA from each cell line was successfully isolated using Trizol reagent (Life Technologies, Carlsbad, CA, USA), and then cDNA was synthesized using M-MLV Reverse Transcriptase (Promega, Beijing, China). After adding the SYBR Premix Ex Taq II (Perfect Real-Time) kit (TaKaRa Bio, Shiga, Japan), qRT-PCR was subsequently carried out with the following settings: 95°C for 30 s and 39 cycles of 95°C for 5 s and 60°C for 30 s. The DNA dissociation analysis (melting curve) was operated at the end of each run to confirm the absence of primer dimers, mixed-amplicon populations, and nonspecific products. The relative expression of genes was presented as comparative threshold cycle (2^-ΔΔCt^) values from at least three independent experiments. Actin Beta (*β-actin*) was used to standardise the expression of target genes. The primer sequences were as follows: *NRP1*, forward 5′- CTTGGCCTGACATTGCAATT-3′ and reverse 5′- AGGTTCCTGCATCCGCCTTAATGT-3′; *FOXP3*, forward 5′- ACTGACCAAGGCTTCATCTGTG-3′ and reverse 5′- GGAACTCTGGGAATGTGCTGTT-3′; *FGF2*, forward 5′- GTCTATCAAAGGAGTGTGTGC-3′ and reverse 5′- TGCCCAGTTCGTTTCAGTG-3′; *NUPR1*, forward 5′- GCGGGCACGAGAGGAAAC-3′ and reverse 5′- CTCAGTCAGCGGGAATAAGTC-3′; *DCBLD2*, forward 5′- ATGTGGACACACTGTACTAGGC-3′ and reverse 5′- CTGTTGGGATAGGTCTGTGG-3′; *β-actin*, forward 5′- AAACGTGCTGCTGACCGAG -3′ and reverse 5′- TAGCACAGCCTGGATAGCAAC′.

### Protein Extraction and Western Blot

Total protein was extracted from cell lines using radioimmunoprecipitation assay lysis buffer (Beyotime, Shanghai, China). Next, the lysates were centrifuged at 12,000 rpm for 30 min at 4°C. The protein concentrations of the lysates were measured using the BCA Protein Assay kit (Genechem, Shanghai, China). Equal amounts of protein (60 µg/lane) were separated by 10% sodium dodecyl sulphate-polyacrylamide gel electrophoresis and then transferred onto PVDF membranes with a pore size of 0.45 µm (Millipore, Billerica, MA, USA). After blocking the membranes with 5% skim milk in TBST at 25°C for 60 mins, the membranes were incubated at 4°C overnight with the following primary antibodies at the stated dilutions: NRP1 (1:1000, Cell Signaling Technology (CST) Shanghai Biological Reagents Company Limited, Shanghai, China), baculoviral IAP repeat containing (BIRC) 3 (1:600, CST), cyclin dependent kinase (CDK) 6 (1:800, CST), Cyclin E (CCNE) 2 (1:800, CST), AP-1 transcription factor subunit (FOS) (1:600, CST), CDK2 (1:1000, CST), CDK4 (1:1500, CST), and β-actin (1:800, CST). After washing in TBST, the membranes were further incubated for 2 h with a secondary anti-mouse (1:3000) or anti-rabbit (1:4000) antibody, as appropriate. Finally, the presentation of target protein bands was enhanced using chemiluminescence (Millipore). The expression levels of target proteins were quantified by densitometry (BioRad image analysis programme) and normalised with respect to β-actin levels.

### Lentivirus-Mediated RNA Interference

Interfering RNAs were delivered by transfection of T24 and 5637 cells with lentivirus vector (GV118, Genechem, Shanghai, China) packaging plasmids containing short hairpin RNAs (shRNAs).To decrease the levels of endogenous *NRP1* or *NUPR1*, *NRP1* specific shRNAs (shNRP1) or *NUPR1* specific shRNAs (shNUPR1) were cloned into GV118 lentivirus vector, and shNRP1 lentivirus 3.30μl (3E+8 TU/ml), or shNUPR1 lentivirus 3.30μl (7E+8 TU/ml), or negative control shRNAs lentivirus 1.00μl (1E+9 TU/ml) were added into each well (5×10^4^ cells per well in 6-well plates) in the presence of 5μg/mL polybrene. Forty-eight hours after infection, cells expressing shRNA were selected using 0.5mg/mL puromycin for 10 days. qRT-PCR was used to test the expression of *NRP1* or *NUPR1* in infected cells. The target sequence of shNRP1-1 was 5′-GCCTTGAATGCACTTATAT-3′, that of shNRP1-2 was 5′-GACCCATACCAGAGAATTA-3′, and that of shNRP1-3 was 5′-AACGATAAATGTGGCGATA-3′. The sequence of the control shRNAs was 5′-TTCTCCGAACGTGTCACGT-3′. The sequence of shNUPR1-1 was 5′-CCAAGCTGCAGAATTCAGA-3′.

### MTT Assay

Cells were seeded in 96-well cell culture plates at an initial density of 0.2 × 10^4^ cells/well in triplicate at a volume of 200 µL/well. According to the experimental requirements, cells were incubated with 100 μL of 0.5 mg/mL sterile 3-(4, 5-dimethyl-2-thiazolyl)-2,5-diphenyl-2H-tetrazolium bromide (MTT; Sigma, USA) at 37°C for different time points. After 4 h, the culture medium was removed and 150 μL of DMSO (Sigma) was added to each well for 10 min to fully dissolve the crystals. Finally, we measured the absorbance values of each well at 490 nm with 570 nm as the reference wavelength to generate the growth curve.

### Colony Formation Assay

Cells were cultured in 60-mm plates at a density of 0.5 × 10^3^ cells/plate for 14 days. The culture medium was then removed. The cells were carefully washed with phosphate-buffered saline (PBS) twice and subsequently fixed with 10% formaldehyde for 5 min, which was followed by staining with 1% crystal violet for 30s. The stain was washed away slowly with running water and the plates were dried at room temperature before counting the number of colonies.

### Tube Formation Assay

A volume of 200µL precooled Matrigel (BD Biosciences, San Jose, CA, USA) was pipetted into wells of a 24-well plate and polymerised at 37°C for 30 min. Subsequently, human umbilical vein endothelial cells (HUVECs) were added to the wells at a density of 0.2 × 10^4^ cells/well in 200µL conditioned medium and incubated at 5% CO_2_ at 37°C for 12 h. Bright-field microscopy at 100× magnification was used to capture the images. The overall length of the complete tubule structures was measured to quantify the capillary tubes.

### Flow Cytometric Apoptosis Test

Cells were digested with 0.25% trypsin, washed with PBS, and centrifuged at 1000 rpm for 5 min. The supernatant was aspirated, and, according to the instructions of the Annexin-V-APC apoptosis determination kit (Ebioscience, USA), we added 100 μl of 1× binding buffer cautiously to each tube. Next, 5 μl of propidium iodide (PI) (Sigma) and 5 μl of Annexin-V-APC were added to the tubes. The tubes were then incubated at room temperature for 15 min, protected from light, before placing on ice. Within 1 h, apoptosis was assessed using the BD FACSCalibur flow cytometer (BD Biosciences).

### Flow Cytometry Cell Cycle Analysis

Cells were digested with 0.25% trypsin, washed with PBS, and centrifuged at 1000 rpm for 5 min. The cell pellet was washed twice with PBS, and resuspended in 0.5 mL of PBS. The tubes were oscillated on a low-speed oscillator, and 70% ice-cold ethanol was added to fix the cells overnight at 4°C. The fixed cells were subsequently centrifuged at 1000 rpm for 5 min. The supernatant was discarded, and the pellet was washed with PBS and resuspended. Bovine pancreatic RNase (Fermentas, Lithuania) was added at a final concentration of 2 mg/mL and the tubes were incubated in a 37°C water bath for 30 min. PI was added at a final concentration of 65 µg/mL, followed by incubation in an ice bath for 30 min protected from light. Finally, cell cycle detection and data analysis were performed using a BD FACSCalibur flow cytometer filtration and FLOWJO Software (Tree Star, Inc, Ashland, OR, USA).

### Transwell Cell Migration Assay

Cells in the logarithmic growth stage were digested, centrifuged and resuspended in serum-free medium. A volume of 750 µL culture medium with serum was added to the bottom of a 24-well plate, and migration chambers were placed in the wells. We added 600 µL of 30% serum-free medium to each chamber and added 100 µL of cell suspension at a density of 1 × 10^5^ cells/mL. After incubation at 37°C for 24 h, the medium was removed from the chambers, and the wells were washed twice with PBS. Migrated cells were fixed by formaldehyde for 30 min before a 15-min staining with Giemsa stain, followed by washing twice with PBS. The non-migrated cells in the bottom of the chamber were scraped off with cotton swabs. Migrated cells were counted in three random fields of view using a light microscope (200×), and images were captured.

### Transwell Cell Invasion Assay

Matrigel was diluted using serum-free medium and mixed well by pipet. A volume of 100 µL prepared Matrigel was added to Transwell chambers in a 24-well plate and incubated at 37°C overnight for gelling. Cells in the logarithmic growth stage were digested, centrifuged, and resuspended in serum-free medium. A volume of 500 µL cell suspension at a density of 1 × 10^5^ cells/mL was placed in the chamber. We subsequently added 750 µL culture medium with serum in the bottom of the wells of a 24-well plate and placed the Transwell chambers into the wells. After incubation at 37°C for 12 h, the medium was removed from the chambers, and the wells were washed twice with PBS. The invasive cells were fixed by formaldehyde at room temperature for 30 min, followed by a 15-min staining with Giemsa stain, and then washed twice with PBS. The non-invasive cells on the bottom of the chamber were scraped off with cotton swabs. Invasive cells were counted in three random fields of view using a light microscope (200×), and images were captured.

### Affymetrix Gene Expression Profile Chip Detection

We extracted total RNA from normal control cells and *NRP1*-knockdown cells with TRIZOL reagent as described above and quantified RNA using the NanoDrop ND-2000 (Thermo Scientific, USA). RNA integrity was further analysed using the Agilent Bioanalyzer 2100 (Agilent Technologies, USA). cDNA libraries were constructed after confirming RNA purity (A260/A280: 1.7-2.2) and RNA integrity (RNA integrity number ≥7.0). Total RNA was transcribed to double-stranded cDNA and synthesised to cRNA. In this process, 2nd-cycle cDNAs were generated and further hybridised onto the microarray after fragmentation and biotin labelling. Microarrays were washed and stained on the GeneChip Fluidics Station 450, and subsequent scanning was performed using the GeneChip Scanner 3000 (Affymetrix, USA). The genes with fold change ≥2.0 and P<0.05 were considered significantly differentially expressed genes (SDEGs).

### GO and KEGG Enrichment Analyses

GO analysis is a commonly-used approach for annotating genes and gene products with their molecular functions and associated biological pathways and cellular components (15). KEGG is a useful resource for the systematic analysis of gene functions and related high-level genome functional information (16). In this paper, the DOSE (17) and clusterProfiler (18) packages of the statistical software R (Version 3.6.3) were used for mining information related to the biological effects of differential expressed genes and implementing pathway enrichment. Subsequently, the ggplot2 and pROC packages were used for high-quality graph generation. GSEA is a computational method that determines whether a previously-defined set of genes shows statistically significant, concordant differences between two biological states (19). GSEA4.0.3 was used for GSEA analysis. The functional gene set file “c2.cp.kegg.v7.0.symbols.gmt” was used to summarise specific and well-defined signaling. The number of substitutions per analysis was set at 1,000, and gene sets with *p* < 0.05 were recognised as significantly enriched.

### Analysis of Gene Expression Profiles by Ingenuity^®^ Pathway Analysis (IPA^®^)

The DEGs were analysed by IPA^®^, which can predict downstream effects and identify new targets or candidate biomarkers and can obtain data analysis and interpretation to understand the experimental results within the context of biological systems. IPA^®^ data analysis is divided into five modules: canonical pathway analysis, disease and function analysis, upstream analysis, molecular interaction network analysis, and regulator effects analysis.

### Construction of the Competing Endogenous RNA (ceRNA) Sankey Diagram

To further analyse the potential regulators of the hub genes, we established a ceRNA network. miRNAs related to NRP1 were predicted in TargetScan. Then, using the edgeR package in the R statistical environment, significant differentially expressed long non-coding RNAs (DElncRNAs) were identified in 411 BC and 19 adjacent non-cancer bladder tissues from TCGA database. | Log2FC | > 2.0 and FDR adjusted to *p* < 0.05 were set as the thresholds. Besides, the significant differentially expression miRNAs (DEmiRNAs) were identified with the thresholds of |Log2 FC| > 1.0 and adj. *p*-value < 0.05 in 415 BC and 19 adjacent non-cancer bladder tissues from the TCGA database. Using miRcode (http://www.mircode.org/), the DElncRNA related DEmiRNA was predicted, while the DEmiRNA with different regulated trend to both NRP1 and lncRNAs were reserved. Finally, the ceRNA network was sankey diagram, which was visualised using dycharts online platform (https://dycharts.com).

### Statistical Analysis

All statistical analyses were conducted using SAS 9.43 statistical software (SAS Institute Inc., Cary, NC, USA). One-way ANOVA was carried out to perform significance tests on the data groups. Significant differences in continuous data (mean ± standard deviation) were evaluated using the Student’s *t*-test. A *p* < 0.05 was considered to be statistically significant.

## Results

### NRP1 Is Upregulated in BC

**Figure 1:**
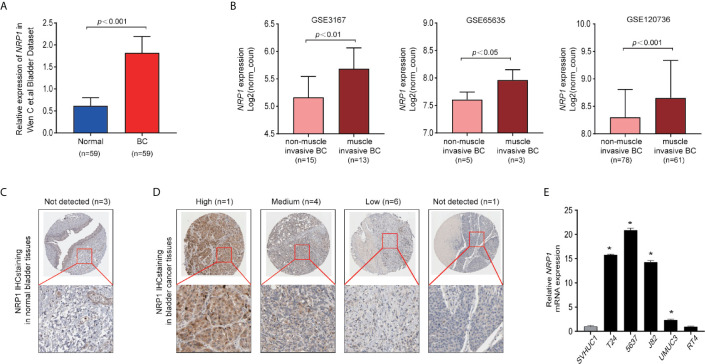
NRP1 is upregulated in BC. **(A)** NRP1 was overexpressed in BC tissues (n = 59) compared with that in normal bladder tissues (n = 59) in the Wen C et al. bladder dataset. **(B)** Expression of *NRP1* was upregulated in muscle invasive BC tissues compared with that in non-muscle invasive BC tissue samples in the GSE3167, GSE65635, and GSE120736 datasets, respectively. **(C)** Representative IHC images of NRP1 in normal bladder tissues and in **(D)** BC tissues. **(E)** The levels of the *NRP1* mRNA in SVHUC1 cell line and five BC cell lines examined using real-time PCR. The average *NRP1* mRNA expression was normalised to the expression of β-actin. Three independent experiments were conducted for each assay and *p < 0.01 *vs*. the SVHUC1 group.

The expression of NRP1 was previously shown to be significantly higher in BC samples compared to adjacent noncancerous tissues (7) (**Figure 1A**). Analysis of the expression of *NRP1* in published profiles (9–11) from MIBC patients showed a frequent upregulation compared to NMIBC tissues (**Figure 1B**). Furthermore, IHC staining data from the Human Protein Atlas (HPA) database was retrieved to confirm the expression of NRP1 protein. While the NRP1 staining in normal bladder tissues was generally not detected, a high proportion of the BC tissues displayed high (1/12), moderate (4/12) or low (6/12) NRP1 staining, which was typically located in the cytoplasm and membrane of cancer cells (**Figures 1C, D**). The qRT-PCR was employed to assess *NRP1* mRNA expression in BC cell lines, and a significant advancement in T24, 5637, J82 and UMUC3 cells comparing to human SVHUC1 cell was observed (**Figure 1E**).

### NRP1 Modulates BC Cells Proliferation and Angiogenesis

**Figure 2:**
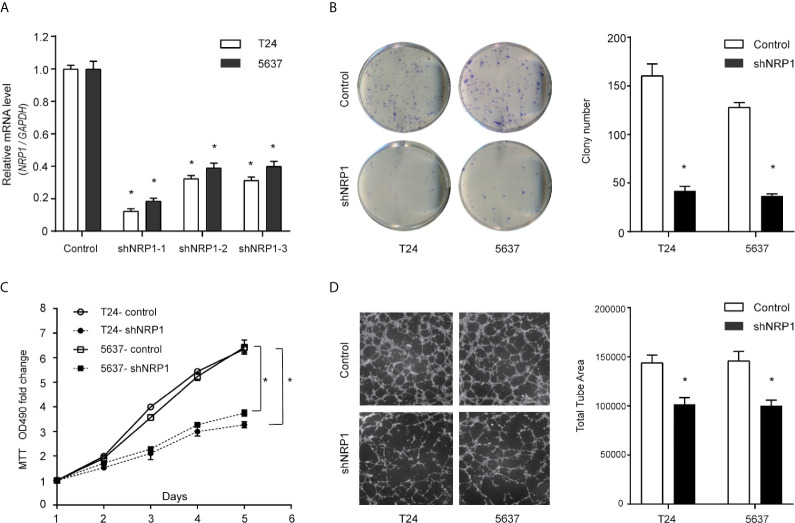
Downregulation of NRP1 reduces BC cells proliferation and angiogenesis. **(A)** T24 and 5637 cells were infected with lentivirus-expressing *NRP1* shRNA-1, shRNA-2, and shRNA-3, or a control shRNA; the *NRP1* mRNA level as measured using qRT-PCR. **(B)** Downregulation of NRP1 reduced the mean colony number in the colony formation assay. **(C)** MTT assays revealed that downregulation of NRP1 significantly reduced the growth rate of BC cells. **(D)** Downregulation of NRP1 reduced tubule formation of vascular endothelial cell. Three independent experiments were conducted for each assay, and data are presented as the mean ± standard error of the mean, *p < 0.01 *vs*. the control group.

Transfection efficiencies of shNRP1s in T24 and 5637 cells were 87.6% and 81.4% (shNRP1-1), 67.6% and 60.9% (shNRP1-2), 68.6.0% and 60.0% (shNRP1-3), respectively (**Figure 2A**), therefore shNRP1-1 was selected to be used in subsequent functional studies. In colony formation assays, *NRP1* knockdown caused a significant reduction in colony number in both T24 and 5637 BC cells (*p* < 0.05 for both; **Figure 2B**). Additionally, MTT assays indicated that *NRP1* knockdown significantly inhibited growth of T24 and 5637 cells, and compared to control cells, the growth rate decreased by nearly 2.0-fold after 5 days (**Figure 2C**). Further, conditioned medium from shNRP1 T24 or 5637 cells was able to significantly suppress tubule formation by HUVECs (*p* < 0.05 for both; **Figure 2D**). These results demonstrate that NRP1 may play a role in promoting proliferation and angiogenesis in BC.

### Silencing NRP1 Promotes BC Cell Apoptosis and Cell Cycle Arrest

**Figure 3:**
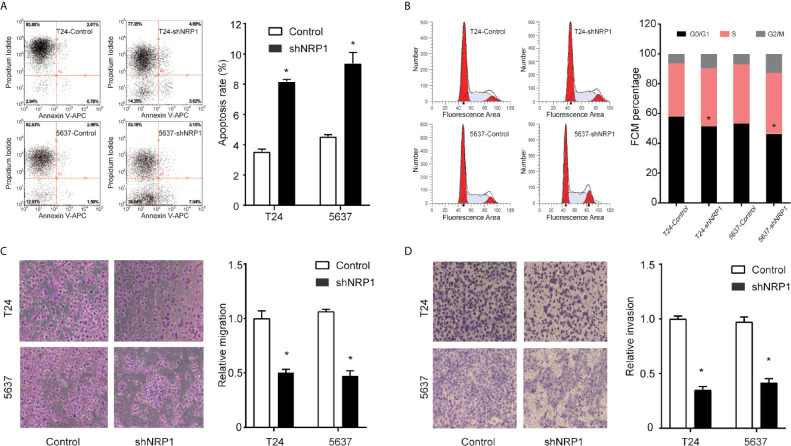
NRP1 modulates BC cells apoptosis, cell cycle, migration, and invasion. **(A)** Apoptosis assay and quantitation of apoptotic cells of T24 and 5637 cells after *NRP1* knockdown or control shRNA expression. **(B)** Flow cytometric analysis of T24 and 5637 cells following *NRP1* knockdown or control shRNA expression. **(C, D)** Images and normalised migration **(C)** or invasion **(D)** of T24 and 5637 cells following *NRP1* knockdown or control shRNA expression. Three independent experiments were conducted for each assay, and data are presented as the mean ± standard error of the mean, *p < 0.05 *vs*. the control group.

To explore the possible mechanism of the proliferation-promoting function of NRP1, apoptosis was examined in *NRP1*-knockdown cells. Silencing *NRP1* increased the proportion of apoptotic cells compared to control cells (**Figure 3A**). Moreover, cell cycle arrest serves as a primary mechanism for inducing apoptosis and flow cytometry analysis showed that *NRP1* knockdown caused a significant decrease in the percentage of cells in the G0/G1 peak, and an increase in the percentage of cells in the G2/M peak, however statistically significant changes were not observed in the S peak (**Figure 3B**), indicating that NRP1 may promote proliferation in BC cells by reducing apoptosis through mediating the G0/G1 and G2/M phase transitions.

### NRP1 Modulates the Migration and Invasion of BC

To evaluate whether NRP1 affects the process of migration and invasion in BC, we performed Transwell assays in T24 and 5637 cells following *NRP1* knockdown. *NRP1* knockdown significantly weakened the migration and invasion abilities in T24 and 5637 cells (**Figures 3C, D**). Migration and invasion in T24 cells decreased by 51% (*p* < 0.05) and 72% (*p* < 0.05) after *NRP1* knockdown, respectively, and by 61% (*p* < 0.05) and 65% (*p* < 0.05), respectively, in 5637 cells. Our results indicate that silencing NRP1 inhibited the migration and invasion ability of BC cells.

### DEGs Between NRP1 Knockdown and Control Group

**Figure 4:**
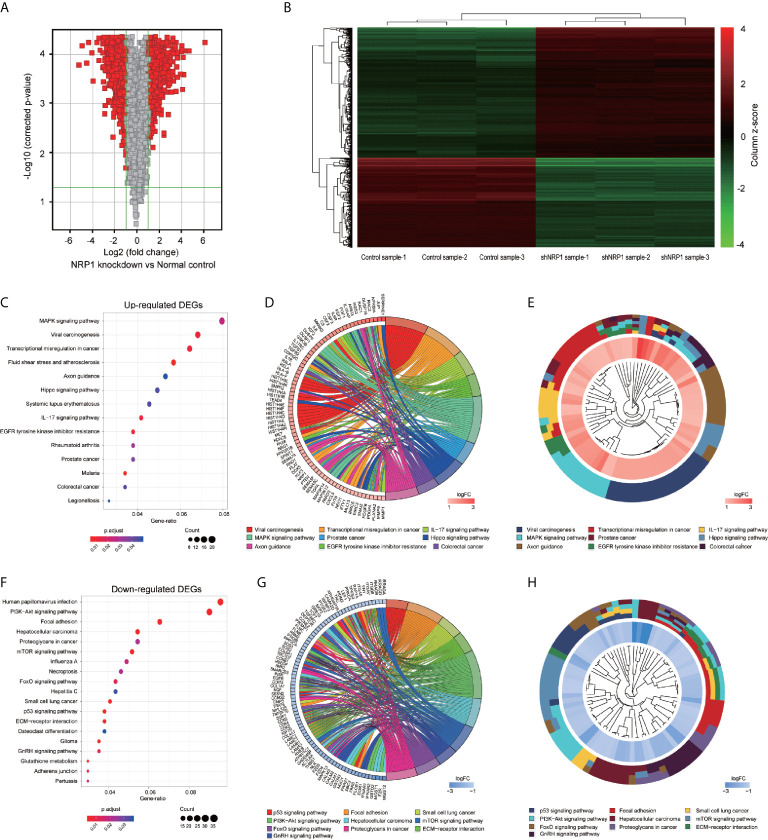
KEGG pathway analysis of DEGs following knockdown of NRP1. **(A)** Gene expression volcano plot of T24 cells transfected with *NRP1* shRNA and a control shRNA vector. The red color on the left side represents 599 upregulated genes, and that on the right side represents 880 downregulated genes (log_2_FC> 2 and *p*-value < 0.05). **(B)** Heatmap and hierarchical cluster analysis of T24 cells transfected with *NRP1* shRNA and a control shRNA vector. Column represents sample, and row represents gene, green represents a lower level gene expression, and red represents a relatively higher of gene expression. **(C)** Bubble plot of KEGG pathway analysis of upregulated DEGs. KEGG pathway description was assigned to y-axis and gene ratio was assigned to horizontal axis as the proportion of differential genes in the whole gene set. The dot size represents the gene counts in a certain pathway. **(D)** KEGG Chord plot of the relationship between the enrichment pathways and their corresponding genes in the upregulated DEGs list. A gene was linked to a certain pathway by the colored bands, and blue-to-red coding next to the genes indicates log FC. **(E)** KEGG Cluster of the upregulated DEG grouped by their functional categories. The inner ring shows the color-coded logFC, and the outer ring represents the assigned signaling pathways. **(F)** Bubble plot, **(G)** KEGG Chord plot, and **(H)** KEGG Cluster plot of KEGG pathway analysis of downregulated DEGs.

To better understand the potential molecular mechanisms underlying BC malignant progression associated with NRP1, we further conducted Affymetrix Gene Chip hybridisation analysis in T24 cells following stable *NRP1* knockdown. After subsequent bioinformatic and normalization analyses, we distinguished the two groups clearly by hierarchical clustering and principal component analyses. According to the microarray expression profiling data, 599 upregulated and 880 downregulated genes had at least 2-fold expression change (*p* < 0.05 for all) following *NRP1* knockdown (**Figures 4A, B**).

### GO Classification and KEGG Pathway Enrichment Analysis of DEGs

GO classification analyses of the upregulated and downregulated DEGs induced by *NRP1* silencing were performed, and a total of 405 and 166 remarkably (adj. *p*-value ≤ 0.05) enriched GO terms including biological process (BP), cellular component (CC) and molecular function (MF) were obtained, respectively. The upregulated DEGs were primarily involved in epigenetically related biological processes, such as regulation of DNA packaging, chromatin silencing, cell differentiation, nuclear and cell division. The downregulated genes were mainly enriched in the regulation of metabolic process, cell communication and cellular response to multiple factors (**Supplementary Table 2**). **Supplementary Figures 1A, B** represents the prior significantly enriched GO terms of upregulated and downregulated DEGs in each classification in the bubble graphs. The results of KEGG pathway analysis (**Supplementary Table 3**) indicated that the upregulated DEGs were significantly enriched in 14 terms, such as mitogen-activated protein kinase (MAPK) signaling, IL-17 signaling, Hippo signaling pathway, Transcriptional misregulation in cancer, and EGFR tyrosine kinase inhibitor resistance (**Figure 4C**); and the downregulated DEGs were remarkably enriched in 19 terms, such as P53 signaling, PI3K-AKT signaling, ECM-receptor interaction, mTOR signaling pathway, etc. (**Figure 4F**). The relationship between the selected pathways and their corresponding genes, and the clustering of the expression profiles were displayed in the chord plot (**Figures 4D, E**) and circular dendrogram (**Figures 4G, H**).

### IPA^®^ Bioinformatics Analysis-Canonical Pathway Analysis

**Figure 5:**
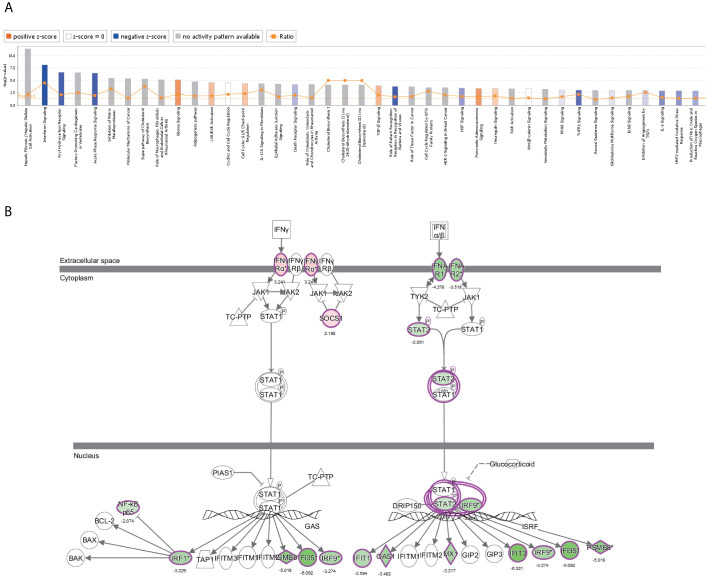
The canonical pathway analysis by IPA^®^. **(A)** The enrichment of the DEGs in the canonical signaling pathway, sorted by –log(P). **(B)** The effect of experimental data on signal transfer in the interferon signaling pathway.

The canonical pathway analysis by IPA^®^ shows for enrichment of the DEGs in the canonical signaling pathway. Our analysis revealed highly significant overlap of 398 canonical pathways (*p* < 0.05) related to tumorigenesis and tumor progression (**Supplementary Table 4**). Interferon signaling (-log P = 8.1, z-score = -2.714), JAK/STAT signaling (-log P = 1.39, z-score = -3),ERK/MAPK signaling (-log P = 1.49, z-score=1.606), p53 signaling (-log P = 2.84, z-score = 1.732), Toll-like receptor signaling (-log P = 2.13, z-score = 1.667), NF-κB signaling (-log P = 2.1, z-score = 1.342), cell cycle (-log P = 4.35, z-score = 0.632), and TGF-β signaling (-log P = 3.92, z-score = 0.775) were affected by *NRP1 k*nockdown in BC T24 cells (**Figure 5A**). Z-score > 0 indicates that the pathway is activated and z-score < 0 indicates that it is inhibited. Among these pathways, interferon signaling was the top enriched signaling pathway ranked in the |z-score| > 2. The effect of DEGs on signal transfer in the interferon signaling pathway are demonstrated in **Figure 5B**.

### NRP1 Is Associated With the Molecular Mechanisms of Cancer Pathways

**Figure 6:**
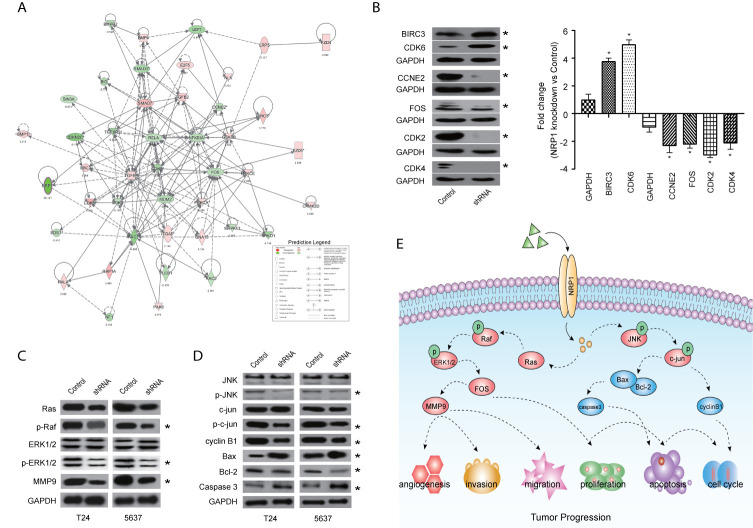
Validation of pathways associated with NRP1 silencing. **(A)** The gene network map of molecular mechanisms of cancer pathway including the potential NRP1-regulated genes. **(B)** The protein expression of some known tumor-associated genes in molecular mechanisms of cancer pathway were confirmed using western blot in T24 cells with *NRP1* knockdown. Western blot was performed in three independent experiments and independently represent each internal control (GAPDH). **(C)** Western blot of ERK/MAPK related protein expression in T24 and 5637 BC cells after *NRP1* knockdown. **(D)** Western blot of JNK/MAPK related protein expression in T24 and 5637 BC cells after *NRP1* knockdown. **(E)** Proposed model for the molecular mechanisms underlying the action of NRP1 in BC progression. *p < 0.05 vs. the control group.

Among these significantly activated pathways, the molecular mechanisms of cancer pathway was chosen to examine the potential role of NRP1 in BC (**Supplementary Table 5**). A gene interaction network in this pathway was constructed to identify the potential NRP1-regulated genes, and NRP1 was presumed to influence the development of BC by regulating the expression of these genes (**Figure 6A**). Western blot was further performed to confirm the dysregulation of certain known tumor-associated genes in T24 cells with *NRP1* knockdown. Consistent with gene chip analysis results, the protein expression of BIRC3 and CDK6 were significantly upregulated following *NRP1* knockdown, while CDK4, CCNE2, FOS, and CDK2 were significantly downregulated (**Figure 6B**).

### NRP1 Is Associated With the MAPK Signaling Pathway

According to the results of KEGG pathway and IPA^®^ canonical pathway analyses of the altered gene sets, we found that the DEGs following *NRP1* knockdown were significantly associated with activation of MAPK signaling pathway. Western blot analysis confirmed that NRP1 function was closely associated with the ERK/MAPK and MAPK8 (JNK)/MAPK signaling. Moreover, Ras, phospho-Raf (p-Raf), p-ERK1/2, and matrix metallopeptidase 9 (MMP9) were all decreased in *NRP1*-knockdown cells (**Figure 6C**), indicating that ERK/MAPK pathway activation is modulated by NRP1. Further, the expression of JNK/MAPK signaling-related key proteins, such as p-JNK, p-c-jun, and cyclin B1, were significantly lower in *NRP1*-knockdown cells (**Figure 6D**), however, the expressions of BCL2-associated X protein (BAX)/BCL2 apoptosis regulator (BCL2) and caspase 3 were higher, which was consistent with bioinformatics signaling enrichment assays. These results suggest NRP1 as an effect factor of MAPK signaling that contributes to cell cycle modulation and drives tumorigenesis in BC (**Figure 6E**).

### IPA^®^ Bioinformatics Analysis-Diseases and Functional Analysis

**Figure 7:**
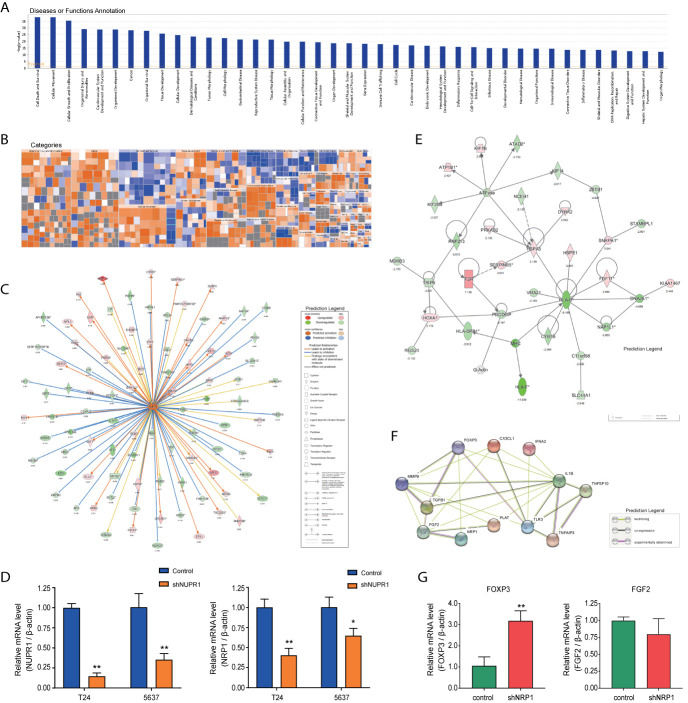
Disease and functional, upstream and network analysis by IPA^®^. **(A)** Disease and functional analysis using IPA^®^ evaluated the positive or negative correlation between NRP1 and other diseases or functions, which were ranked by log (*p*-value). **(B)** The heatmap demonstrates the relationship between DEGs expression and activation or inhibition of diseases and functions categories. **(C)** Upstream analysis predicted that the NUPR1 regulatory network was activated after *NRP1* was knocked down. **(D)** T24 and 5637 cells were infected with lentivirus-expressing NUPR1 or a control shRNA; the *NUPR1* and *NRP1* mRNA level was measured using qRT-PCR. **(E)** The molecular interaction network analysis predicted interaction among the molecules in the dataset and found that the top-ranked molecular interaction network was primarily enriched in the diseases and function categories of cancer, organismal injury and abnormalities and cell cycle, which including the altered genes after *NRP1* silencing are shown. **(F)** The regulatory effect network analysis revealed that NRP1 may be a regulator in neovascularisation activation. **(G)** the mRNA expression of *FOXP3 and FGF2* gene in 5637 cells with *NRP1* silencing were measured using qRT-PCR. *p < 0.05 *vs*. the control group. **p < 0.01 *vs*. the control group.

Disease and functional analysis by IPA^®^ evaluated the positive or negative correlation between NRP1 and other diseases or functions (**Figure 7A**). The annotation of diseases or functions with significant activation were cancer (z-score = 2.568), proliferation of tumor cells (z-score = 2.479), migration of endothelial cells (z-score = 2.535), cell movement of endothelial cells (z-score = 2.354), neovascularization (z-score = 2.073), etc. Alternatively, antiviral response (z-score = -3.213), immune response of cells (z-score = -2.974) and G1 phase (z-score = -2.176) were significantly inhibited (**Supplementary Table 6**). NRP1 silencing was related to many cancer-related functions, which is consistent with the results of cell function experiments. The heatmap demonstrates the relationship between DEG expression and activation or inhibition of diseases and function categories (**Figure 7B**). Antiviral response (z-score = -3.213) was the most significantly affected annotation sorted by | z-score| (**Supplementary Figure 2**).

### IPA^®^ Bioinformatics Analysis-Upstream Analysis and Validation

Analysis was performed to predict the upstream regulatory factors of DEGs (**Supplementary Table 7**. The predictive interactions were supported by literature based on the Ingenuity Pathway Knowledge Base (IPKB). IPA predicted upstream regulators related to tumorigenesis that were contained in the DEGs list, such as HIF1α (z-score = 2.376, overlap *p*-value = 1.2E-11), TGFβ (z-score = 4.222, overlap *p*-value = 1.181E-44), MAPK1 (z-score = 4.399, overlap *p*-value = 2.73E-29). Moreover, nuclear protein 1 (*NUPR1*) was predicted to be most strongly activated, with 74 consistent activated DEGs, while IFNB1 was predicted to be the most strongly repressed, with 37 consistent repressions. The *NUPR1* regulatory network containing the interacting genes in the DGEs list was presented in **Figure 7C**. The DEGs following *NRP1* silencing are primarily downstream of *NUPR1* in BC cells. Further validation by qRT-PCR found that *NRP1* was significantly downregulated when *NURP1* was knocked down (transfection efficiencies of shNUPN1 were respectively 85.3% and 64.6% inT24 and 5637 cells) (**Figure 7D**), suggesting that *NRP1* was regulated by *NUPR1*.

### IPA^®^ Bioinformatics Analysis-Molecular Interaction Network Analysis

IPA^®^ uses a network generation algorithm to segment the molecular interaction network into multiple networks and scores each network. The score is based on the hypergeometric distribution, and the -log(P) value was obtained by Fisher’s exact test. The top-ranked molecular interaction network was primarily enriched in diseases and functions related to cancer, organismal injury as well as abnormalities and cell cycle (**Supplementary Table 8**). The network including the altered genes following *NRP1* silencing are presented in **Figure 7E**.

### IPA^®^ Bioinformatics Analysis-Regulator Effects

The regulatory effect network analysis shows the interaction between genes and regulators and functions in the IPKB (**Supplementary Table 9**). The consistency score is a measure of causal consistency and dense connections between upstream regulatory factors and diseases and functions in IPKB. The higher the consistency score is, the more accurate the results of the regulatory effects prediction. The result of regulator analysis shows that *CX3CL1*, *FOXP3*, and *IFNA2* act as regulators through *FGF2, IL1B, MMP9, NRP1, PLAT, TGFB1, TLR3, TNFAIP3*, and *TNFSF10* to activate neovascularization; while NRP1 was predicted to bind with FOXP3 and FGF2 directly (**Figure 7F**). Further qRT-PCR detection showed that after *NRP1* silencing *FOXP3* was significantly upregulated, but *FGF2* gene was minor downregulated insignificantly (**Figure 7G**).

### Analyses of *NRP1* in BC Cells *via* CCLE

**Figure 8:**
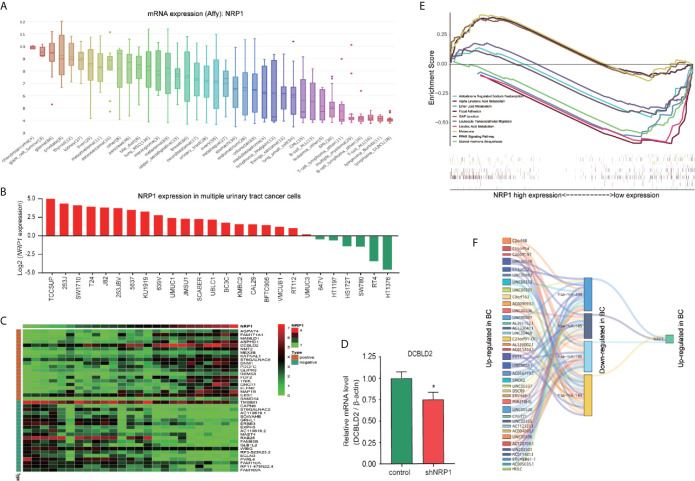
Co-expressed genes analysis and ceRNA network prediction. **(A)** *NRP1* mRNA expression in various cancer cell lines obtained from CCLE database. The abscissa is the tumor type and sample size, and the ordinate is the expression of target genes. **(B)** *NRP1* mRNA expression in 26 urinary tract cancer cells extracted from CCLE database. We took the logarithm *of* the original data for better visualization. **(C)** Heatmap of the top 20 co-expressed genes with *NRP1* in urinary tract cancer cells in both upregulated and downregulated groups. **(D)** the mRNA expression of *DCBLD2* in 5637 cells with *NRP1* silencing were measured using qRT-PCR. *p < 0.05 *vs*. the control group. **(E)** The significantly enriched signal transduction pathways of co-expressed genes of *NRP1* in urinary tract cancer cells obtained using GSEA analysis. **(F)** A ceRNA sankey diagram of NRP1 constructed using the data extracted from TCGA database.

The expression of *NRP1* can be detected in a variety of malignant cells (**Figure 8A**) and urinary tract cancer cells (**Figure 8B**) in Cancer Cell Line Encyclopedia (CCLE) database. Using the co-expression tool on expression data extracted from the 26 urinary tract cancer cell samples, we obtained lists of genes that are co-expressed with *NRP1*. Genes that harbor a correlation coefficient > 0.5 or <-0.5, and *p*-value <0.01 were selected. A total of 445 genes were positively and 433 were negatively correlated with *NRP1* expression (**Supplementary Table 10**). The expression data for the top 20 related up-and downregulated groups were depicted in heatmaps (**Figure 8C**). Notably, in the positive correlation list, *DCBLD2* was highly correlated with *NRP1* of 0.775, while the fold change of *DCBLD2* was -4.917 in T24 cells after *NRP1* knockdown by Gene Chip analysis, and *DCBLD2* was also found by qRT-PCR significantly downregulated by 0.751 Log2 fold in 5637 cells with *NRP1* silencing (**Figure 8D**). To identify the differentially activated signaling pathways in BC cells, Gene Set Enrichment Analysis (GSEA) was performed and the most significantly enriched signal transduction pathways were selected (**Figure 8E** and **Supplementary Table 11**). Focal adhesion, melanoma, and GAP junction were differentially enriched in phenotypes with high *NRP1* expression, while Peroxisome proliferator-activated receptor (PPAR) signaling and multiple metabolism-related pathways were significantly enriched in low *NRP1* expression phenotypes.

### Construction of the ceRNA Sankey Diagram

Significant differential expressed lncRNA (DElncRNA), and differential expressed miRNA (DEmiRNA) between BC and adjacent non-cancer bladder tissues in the The Cancer Genome Atlas (TCGA) database were identified. A total of 1116 DElncRNAs (761 upregulated and 355 downregulated), and 368 DEmiRNAs (307 upregulated and 61 downregulated) were obtained (**Supplementary Figure 3** and **Supplementary Table 12**). Besides, miRNAs related to NRP1 were predicted in TargetScan. After selecting the miRNAs with differently regulated trends for both NRP1 and lncRNAs, a ceRNA Sankey diagram was constructed, including 38 lncRNA squares, four miRNA squares, and one NRP1 square (**Figure 8F**). NRP1 may be regulated by miR-204, miR-143, miR-145 and miR-195.

## Discussion

NRPs are a class of approximately 130-kDa multifunctional non-tyrosine kinase receptors. The main functional domain of NRPs consists of five domains: one intracellular, one transmembrane, and three extracellular (a1a2, b1b2, and c) domains (20). The membrane domain directly binds to type III semaphorins and VEGF and can initiate downstream signaling. Knockout of NRP1 and NRP2, the two major NRP subtypes, in mice induces hypoplasia and deficiency in the neural system, emphasising their roles in neural development (21). NRP1 is overexpressed in numerous human tumor tissues, including breast, lung, colorectal, and hepatocellular cancer (5, 22). Further, NRP1 expression is positively associated with prostate-specific antigen and Gleason score in prostatic cancer (23), while overexpression may contribute to autocrine-paracrine interactions in pancreatic cancer (24). Herein, high-expressed NRP1 was detected in a variety of BC cell lines by qRT-PCR and human BC tissues by IHC, whose overexpression has been reported to be associated with tumor progression and poor prognosis in patients with BC; however, the underlying molecular mechanisms remain poorly understood (7). Therefore, identifying the mechanisms by which NRP1 modulates the progression of BC may prove significant for exploring and optimising the therapeutic strategy for urological malignancies.

In this study, by comparing the expression of *NRP1* in muscle invasive BC tissues to that in non-muscle invasive BC tissues, we found that increasing *NRP1* was significantly associated with advanced tumor stage. Besides, silencing *NRP1* could promote apoptosis and reduce proliferation, angiogenesis, migration, and invasion in two aggressive BC cell lines. These results clearly identify NRP1 as a tumor promoter in BC and suggest that it has the potential to serve as a target for BC treatment. To better understand how NRP1 drive BC progression, global gene expression profiling using microarray technology and bioinformatics analysis were then performed. According to the enriched KEGG pathway items, the alteration of MAPK signaling contains the largest number of upregulated DEGs. MAPK signaling has been reported to modulate several key biological processes during the development and progression of BC and is regarded as a regulator of cell proliferation, angiogenesis, invasion, and metastasis (25). Ceccarelli et al. reported that NRP1 is responsible for keratinocyte growth factor-dependent ERK/MAPK pathway activation in human adipogenesis (26). Thus, we sought to explore the impact of NRP1 on MAPK pathway activation in BC cells. Key protein markers for MAPK signaling pathway were examined by western blot, and the results indicated that silencing of NRP1 would decrease the activity of phosphorylated ERK and JNK. Additionally, BAX/BCL2 and caspase 3 were upregulated, implying that NRP1 knockdown attenuated anti-apoptotic signals, allowing for the induction of apoptosis. Collectively, these findings suggested that NRP1 could be considered as a vital contributor in BC tumorigenesis and progression through MAPK signaling.

Canonical pathway analysis by IPA^®^ also revealed that all significantly altered genes following *NRP1* knockdown exhibited a significant enrichment in many tumorigenesis- and development-related pathways, which is consistent with the results of KEGG and western blot analysis. Among these pathways, interferon signaling was the top enriched signaling pathway ranked in the |z-score|>2. The expression of interferon-γ receptor was significantly increased after *NRP1* silencing, which is not only used as a therapeutic agent for BC treatment but is activation in bladder tumor cells is required for Bacillus Calmette-Guérin-induced tumor elimination and tumor-specific immune memory (27). Additionally, the molecular mechanisms of cancer pathway were chosen to explore the alteration of tumor-related genes following *NRP1* knockdown in BC. In total, 48 genes were significantly enriched in this pathway. Among these DEGs identified, *CDK6* was the most significantly upregulated gene, and *CDK2* was the most significantly downregulated gene. CDK6 plays an important role in the cell cycle. To drive the progression of the cell cycle, CDK6 binds to, and is activated by, cyclin D to enhance the transition through the G1 phase (28). Wang et al. confirmed that the increased expression of CDK6 was synchronous with the development of BC, indicating that it could be considered a prognostic biomarker for patients with BC (29). Additionally, abnormal CDK6 expression has been detected in breast cancer (30), pancreatic cancer (31), malignant glioma (32), and medulloblastoma (33). Activation of cyclin E/CDK2 and cyclin D1/CDK4 in cell cycle progression could contribute to urothelial proliferation (34), while downregulation of CDK2 in BC was first reported in this study. Collectively, our bioinformatic analysis indicated that NRP1 may influence BC progression through CDK6 and CDK2, as well as BIRC3, CDK4, CCNE2 and FOS, although this requires further validation.

According to the disease and function analysis, we found that *NRP1* knockdown is associated with many malignant tumor-related functions. Among them, antiviral response was the most significantly affected annotation sorted by | z-score|. There is a growing appreciation for roles played by NRP1 in the immune response, especially in the function of regulatory T cell response to virus infection (35, 36). In fact, investigations of the possible correlation between infection with different viruses, including human papilloma virus (HPV), human immunodeficiency virus (HIV), polyomavirus (BK) virus, herpes simplex virus, human T cell lymphotropic virus type 1 (HTLV-1), or Epstein-Barr virus (EBV), and the occurrence of BC are underway (37, 38). As reported, the prevalence of HPV varies greatly in BC cases, while a strong positive association between EBV infection and pathogenesis of primary urothelial transitional cell carcinoma has been found (38). Although only a limited number of BC cases have been linked with HIV infection, BC is part of the growing list of cancers that may be encountered in HIV-infected patients (39). Although the association between NRP1 and HPV remains elusive, HIV could lead to upregulation of NRP1 and suppress the expression of semaphorin 3a in the podocyte (40), while inhibiting VEGF from binding to NRP1 in endothelial cells to block angiogenesis and induce apoptosis (41). Besides, the NRP1 contains domains that directly interact with HTLV-1 (36) and EBV (42). Furthermore, NRP1 is identified as an EBV entry factor, its overexpression enhances EBV infection in nasopharyngeal epithelial cells (42), and highly expressed NRP1 could be consider as an undesirable independent prognostic factor in EBV-associated lymphomas (43). Taken together, the antiviral effect of NRP1 may provide new sight into the understanding of BC therapy.

The regulatory effect network analysis speculated that *NRP1, FGF2, IL1B, MMP9, PLAT, TGFB1, TLR3, TNFAIP3*, and *TNFSF10* might activate neovascularization through interacting with *CX3CL1, FOXP3*, and *IFNA2*, and NRP1 was predicted to directly bind with FGF2 and FOXP3. It has been reported that, in addition to VEGF and semaphorins 3a, NRP1 also specifically binds with several growth factors, including fibroblast growth factor 2 (FGF2), hepatocyte growth factor, platelet derived growth factor, placental growth factor, and transforming growth factor β1 (TGF-β1) (44). FGF2 can promote tumor angiogenesis and metastasis (45, 46). There is also evidence showing that FOXP3 suppresses angiogenesis by inhibiting VEGF expression in breast cancer (47), and on T regulatory cells, FOXP3 contributes to immunosuppression in a NRP1-dependent manner (48). In our study, the results of Gene Chip analysis showed that after *NRP1* silencing *FOXP3* gene was upregulated by 2.188 Log^2^ fold and *FGF2* was downregulated by 1.026 Log^2^ fold. Besides, validation in 5637 cells suggested that when *NRP1* was knocked down *FOXP3* was significantly upregulated, but *FGF2* gene was minor downregulated insignificantly. Our findings are in line with trends found in the literature (45, 47). We therefore speculate that *NRP1* silencing exert anti-angiogenic effects by upregulating *FOXP3* expression. To the best of our knowledge, this is the first time that a negative correlation has been revealed between NRP1 and FOXP3. Certainly, the specific role of NRP1 in FOXP3 as well as FGF2 mediated angiogenesis requires further exploration by biological experiments. On the other hand, cumulating evidence indicates that MAPK signaling activation is associated with VEGF-mediated tumor progression in bladder cancer (49), which was also observed in epidermal cancer stem cells but in a NRP1-dependent manner to enhance angiogenic potential, invasion and migration (50). These findings also coincide with our observations in BC cells that NRP1 silencing lead to the inhibition of angiogenesis and MAPK signaling activity.

The co-expressed genes with *NRP1* in multiple urinary tract cancer cells were obtained, and the corresponding pathways were identified. *DCBLD2*, the co-expressed gene, was also remarkably downregulated by 2.298 Log^2^ fold by Gene Chip analysis in T24 cells after *NRP1* knockdown. Besides, PPAR signaling pathways were significantly enriched in low *NRP1* expression phenotypes. Although no study has directly demonstrated the interaction between DCBLD2 or PPAR signaling and NRP1, there is evidence of a potential association. DCBLD2, a neuropilin-related transmembrane protein expressed in endothelial cells (ECs), promotes endothelial VEGF signaling and regulates EC angiogenesis, proliferation and migration, which may serve as a therapeutic target for angiogenesis regulation. DCBLD2 also associates with VEGFR2 and regulates its complex formation and mediates its trafficking (51). PPAR signaling has a pleiotropic impact on the regulation of cell growth and differentiation, and its role in the angiogenesis suppression is present in a VEGFR2-dependent manner (52).

We also performed upstream analysis to predict upstream regulators of DEGs following *NRP1* silencing, such as HIF1α, TGFβ, and MAPK1, which were all related to tumorigenesis. Notably, NUPR1, which is a transcription factor regulating a complex network of pathways and whose role in various types of cancer including BC has been reported yet remains incompletely understood, was predicted to be most strongly activated (53). NUPR1 participates in the regulation of tumor cell autophagy, apoptosis, growth, migration, and invasion (54); however, no study describing the association with NRP1 has been reported before. Herein, taken together results of Gene Chip analysis that *NUPR1* expression was not detected and *DCBLD2* was significantly downregulated after stable *NRP1* knockdown, it is reasonable to speculate that *NRP1* may be the downstream target of *NUPR1* and essential for regulation of *DCBLD2* expression. Further cell *in vitro* experiments showed a significant decrease of *NRP1* when *NURP1* was knocked down, and a significant decrease of *DCBLD2* after *NRP1* silencing, which demonstrated for the first time that *NRP1* is the downstream target of *NUPR1* and the upstream regulator of *DCBLD2*. Certainly, the specific roles of NUPR1 and DCBLD2 in NRP1 mediated malignant phenotype require further exploration by biological experiments.

Additionally, the ceRNA network analysis results demonstrate that NRP1 may be regulated by miR-204, miR-143, miR-145 and miR-195 in BC. These miRNAs are associated with neovascularisation and all involve VEGF regulation, however, miR-145 has been reported to directly interact with NRP1. miR-145 plays an crucial role in the regulation of interferon-β induction in BC cells (55), and the miR-145-3p/NRP1 axis targeted by the circRNA009723 (circDcbld1) might be a feasible approach to regulate vascular smooth muscle cell migration and alleviate intimal hyperplasia (circDcbld1) (56).

Taken together, these findings provide novel insights into the molecular mechanisms by which NRP1 drives the pathogenesis and progression of cancer. It would be reasonable to believe that targeting NRP1 may be a potential new therapeutic strategy that would be beneficial for more patients with BC or other cancers. Further research into the crucial mechanisms of NRP1 dysregulation in BC development is ongoing to better understand the biological basis of malignancy progression.

In conclusion, we provided evidence for NRP1 expression patterns in BC and found that inhibiting NRP1 expression could promote apoptosis and suppress proliferation, angiogenesis, migration, and invasion of BC cells, implying the potential of NRP1 as an attractive target in BC therapy. We also predicted and confirmed the effect of NRP1 on the activity of MAPK signaling and the dysregulation of genes involved in molecular mechanisms of cancer pathways. *NRP1* silencing also affected various biological functions, including antiviral response, immune response, cell cycle, proliferation and migration of cells, and neovascularisation. In addition, to our knowledge, the association between *NRP1* and *NUPR1, FOXP3*, and *DCBLD2*, for the first time, has been demonstrated. By analysing data extracted from multiple urinary tract cancer cells, PPAR signaling was found significantly associated with low *NRP1* expression. Moreover, NRP1 was predicted to be targeted by miR-204, miR-143, miR-145, and miR-195 in BC development. Further research into the crucial mechanisms of NRP1 dysregulation in BC aggression is also required to improve our understanding of the biological basis of malignancy progression.

## Data Availability Statement

The datasets presented in this study can be found in online repositories. The names of the repository/repositories and accession number(s) can be found in the article/**Supplementary Material**.

## Author Contributions

YD, W-mM, Z-dS, Z-gZ, LH, and C-hH designed this study. YD, W-mM, J-hZ, and LH performed experiments and analysed data. YaL, S-qZ, KP, LX, and B-bL performed bioinformatics analysis. G-yZ, RL, YiL, and C-hH provided technical support. W-dZ and TF performed the statistical analyses. YD, W-mM, and Z-dS drafted the manuscript. Z-gZ, LH, and C-hH provided critical comments, suggestions, and revised the manuscript. Z-dS, LH, and C-hH provided funding support. All authors contributed to the article and approved the submitted version.

## Funding

This work was supported by the National Natural Science Foundation of China [grant numbers 82004110 and 81774089]; the Medical Innovation Team Project of Jiangsu Province [grant number CXTDA-2017-48]; the Key Research and Development Program of Jiangsu Province [grant numbers BE2020758 and BE2019637]; the High-level health talents “Six One Project” top talents [grant number LGY2019058]; the Key Project of Xuzhou Science and Technology [grant numbers KC19075 and KC18036]; and the Outstanding Medical Talent Project of Xuzhou [grant number 22 (2017)].

## Conflict of Interest

The authors declare that the research was conducted in the absence of any commercial or financial relationships that could be construed as a potential conflict of interest.

## Abbreviations

Bax, BCL2-associated X protein; BC, bladder urothelial carcinoma; Bcl2, BCL2 Apoptosis Regulator; BIRC3, baculoviral IAP repeat containing 3; CCNE2, Cyclin E2; CDK, cyclin-dependent kinase; CST, Cell Signaling Technology; DCBLD2, Discoidin CUB And LCCL Domain Containing 2; FOS, AP-1 Transcription Factor Subunit; GAPDH, glyceraldehyde-3-phosphate dehydrogenase; FGF2, Fibroblast Growth Factor 2; FOXP3, Forkhead Box P3; HGF, Hepatocyte Growth Factor; PDGF, Platelet Derived Growth Factor; PGF, Placental Growth Factor; TGF-β1, Transforming Growth Factor β1; HPV, Human Papilloma Virus; HIV, Human Immunodeficiency Virus; HSV, Herpes Simplex Virus; HTLV-1, Human T-cell Lymphotropic Virus Type 1; EBV, Epstein-Barr Virus; NRP1, Neuropilin 1; NUPR1, Nuclear Protein1; GO, Gene Ontology; HUVEC, Human Umbilical Vein Endothelial Cells; JNK, mitogen-activated protein kinase 8; MAPK, Mitogen-Activated Protein Kinase; MMP9, Matrix Metallopeptidase 9; NF-κB, Nuclear Factor Kappa B; NRP, neuropilin; PBS, phosphate-buffered saline; PI, propidium iodide; qRT-PCR, quantitative real-time reverse transcription polymerase chain reaction; SDEG, significantly differentially expressed genes; shRNA, small hairpin RNA; VEGF, vascular endothelial growth factor.
