# Supplementary material for: Role of NRP1 in Bladder Cancer Pathogenesis and Progression
Source: Front Oncol. 2021 Jun 23;11:685980. doi: 10.3389/fonc.2021.685980 (PMC8261128; doi:10.3389/fonc.2021.685980)
Supplement: Supplementary file 1 [file Image_1.pdf]

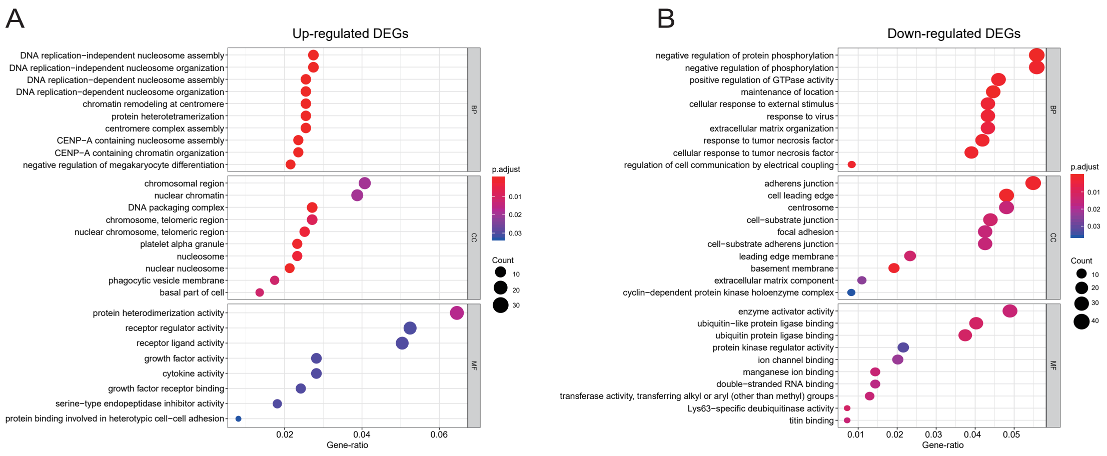

Supplementary Figure 1. GO term classification of DEGs following NRP1 knockdown. (A) Bubble plot of GO terms of upregulated DEGs and **(B)** downregulated DEGs, including biological process (BP), cellular component (CC), and molecular function (MF). GO term description was assigned to y-axis and gene ratio was assigned to horizontal axis as the proportion of differential genes in the whole gene set. The dot size represents the gene counts in a certain GO term.
