# Supplementary material for: Role of NRP1 in Bladder Cancer Pathogenesis and Progression
Source: Front Oncol. 2021 Jun 23;11:685980. doi: 10.3389/fonc.2021.685980 (PMC8261128; doi:10.3389/fonc.2021.685980)
Supplement: Supplementary file 2 [file Image_2.pdf]

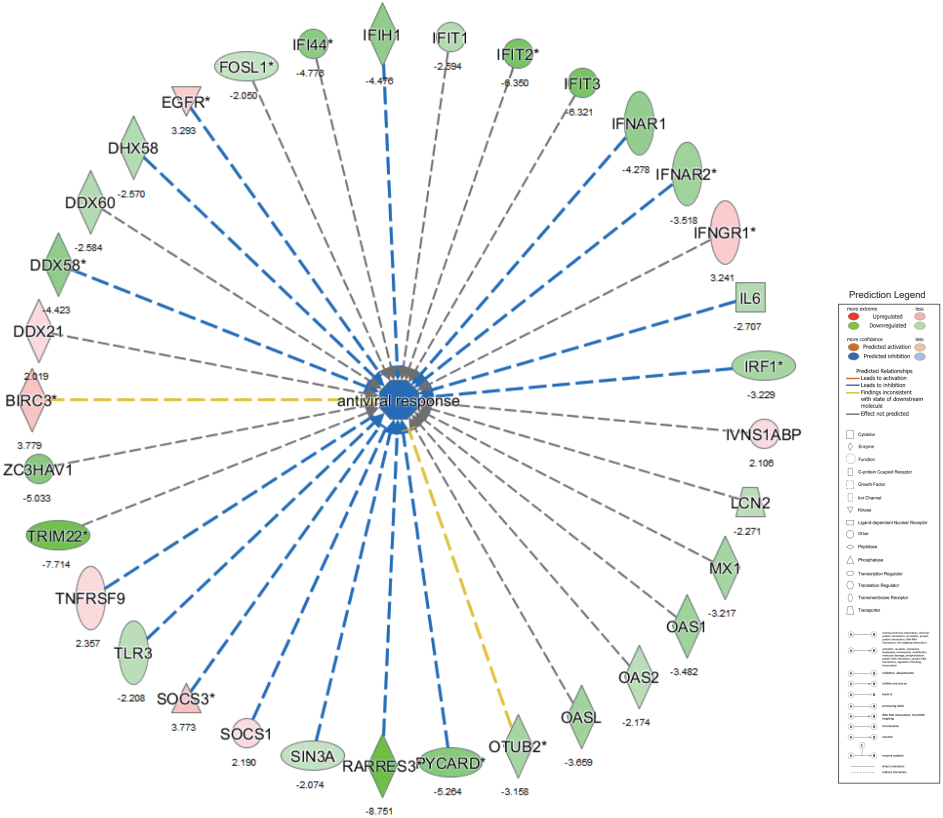

Supplementary Figure 2. Diseases and functional analysis using IPA® - antiviral response. Disease and functional analysis using IPA® revealed that antiviral response was the most significantly affected functional annotation after NRP1 knockdown, and the expression of DEGs involved in this annotation are shown.
