# Supplementary material for: Role of NRP1 in Bladder Cancer Pathogenesis and Progression
Source: Front Oncol. 2021 Jun 23;11:685980. doi: 10.3389/fonc.2021.685980 (PMC8261128; doi:10.3389/fonc.2021.685980)
Supplement: Supplementary file 3 [file Image_3.pdf]

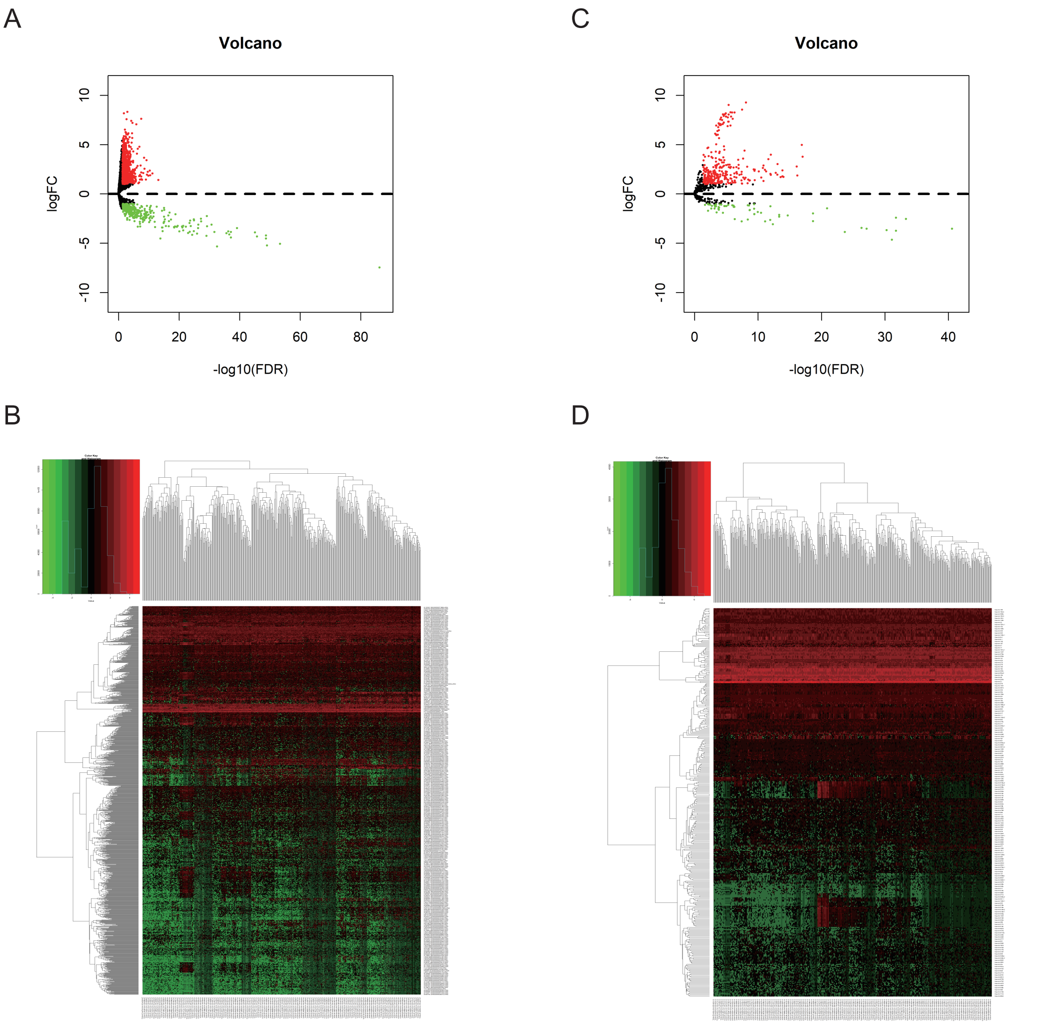

Supplementary Figure 3. DElncRNAs and DEmiRNAs in patients with BC. (A) Volcano plot and **(B)** heatmap of DElncRNAs in BC and adjacent non-carcinoma bladder tissues. The DElncRNAs were identified with the thresholds of |Log2 FC| > 2.0 and adj.P-value < 0.05. **(C)** Volcano plot and **(D)** heatmap of DEmiRNAs in BC and adjacent non-carcinoma bladder tissues. The DEmiRNAs were identified with the thresholds of |Log2 FC| > 1.0 and adj.P-value < 0.05.
