# Supplementary material for: Role of NRP1 in Bladder Cancer Pathogenesis and Progression
Source: Front Oncol. 2021 Jun 23;11:685980. doi: 10.3389/fonc.2021.685980 (PMC8261128; doi:10.3389/fonc.2021.685980)
Supplement: Supplementary file 8 [file Table_5.docx]

**Supplementary Table 5 | The information of molecular mechanisms of cancer pathway.**

| **Ingenuity Canonical Pathway** | **-Log (P value)** | **Ratio** | **Related Molecules in DEGs (Fold Change)** |
| --- | --- | --- | --- |
| Molecular Mechanisms of Cancer | 5.36 | 0.129 | RAC2 (-2.091), RELA (-2.074), BMP4 (2.021), RALA (2.696), SOS2 (-2.432), CDK4 (-2.135), CDKN2C (-3.679), BMPR2 (-2.540), MAPK13 (-2.063), TGFBR2 (-3.562), CAMK2D (2.089), NFKBIA (-2.322), NLK (2.424), BBC3 (3.099), TGFB1 (2.222), SOS1 (-2.144), TGFB2 (3.111), E2F5 (2.457), BID (-2.735), PRKCE (2.080), PLCB1 (-2.439), GSK3B (2400), GNA13 (2.194), BIRC3 (3.779), SMAD1 (-2.73), PRKD1 (-3.749), BMP1 (3.015), PRKCA (2.075), CCNE2 (-2.330), LRP5 (3.137), PAK6 (2.079), CDK6 (4.999), RAC1 (-4.585), SMAD7 (4.572), ITGA5 (2.144), MDM2 (-2.478), RAP1A (4.982), SIN3A (-2.074), ARHGEF5 (-2.728), FOS (-2.223), FZD4 (2.052), NF1 (-3.342), APH1A (-2.386), ARHGEF16 (2.006), FZD5 (2.388), LEF1 (-3.533), CDK2 (-3.005), NRP1 (-5.234). |
